# Activation of the STING pathway induces peripheral sensitization via neuroinflammation in a rat model of bone cancer pain

**DOI:** 10.1007/s00011-022-01663-2

**Published:** 2022-11-08

**Authors:** Yuxin Zhang, Wei Wang, Zhihao Gong, Yuan Peng, Xin Li, Zuojing Zhang, Xiaoxuan Zhang, Xingji You, Jingxiang Wu

**Affiliations:** 1 Department of Anesthesiology, Shanghai Chest Hospital, Shanghai Jiao Tong University School of Medicine, No. 241 Huaihai Road West, Shanghai, 200030 China; 2 School of Medicine, Shanghai University, No. 99 Shangda Road, Baoshan District, Shanghai, 200444 China

**Keywords:** Bone cancer pain, Dorsal root ganglia, STING, Neuroinflammation

## Abstract

**Background:**

Neuroinflammation in the peripheral nervous system has been linked to cancer metastasis-induced bone pain. The stimulator of interferon genes (STING), an innate immune sensor for cytosolic DNA, plays an important role in inflammation and cancer metastasis and is reported to be a critical regulator of nociception. Here, we examined the role of STING in primary nociceptive neurons and chronic pain to determine if it could be a new target for treating bone cancer pain (BCP).

**Methods:**

Walker 256 cancer cells were injected intratibially to induce bone cancer pain in rats. STING and its downstream inflammatory factors in dorsal root ganglia (DRG) were detected using western blotting and immunofluorescent staining. Transmission electron microscopy and the BCL2-associated X (Bax) expression were used to detect the mitochondrial stress in DRG neurons. C-176, a specific inhibitor of STING, was used to block STING activation and to test the pain behavior.

**Results:**

Mechanical hyperalgesia and spontaneous pain were observed in BCP rats, accompanied by the upregulation of the STING expression in the ipsilateral L4-5 DRG neurons which showed significant mitochondrion stress. The STING/TANK-binding kinase 1 (TBK1)/nuclear factor-kappa B (NF-κB) pathway activation was observed in the DRGs of BCP rats as well as increased IL-1β, IL-6, and TNF-α expression. C-176 alleviated bone cancer pain and reduced the STING and its downstream inflammatory pathway.

**Conclusion:**

We provide evidence that STING pathway activation leads to neuroinflammation and peripheral sensitization. Pharmacological blockade of STING may be a promising novel strategy for preventing BCP.

## Introduction

Bone cancer pain (BCP), one of the most severe types of chronic pain, involves the complex interaction of various molecular events, resulting in a combination of inflammatory and neuropathic pain [1–3]. Specifically, peripheral sensitization, which mainly involves persistent afferent stimulation at the site of bone metastasis, inflammatory responses and changes in the plasticity of primary sensory neurons in the dorsal root ganglion (DRG), plays an essential role in the occurrence and development of BCP [4]. Pain information processing starts from activation of peripheral nociceptors in the DRG, and DRG neurons act as a bridge between the internal and external environments. Aside from being traditional sensors for hazardous stimuli, peripheral nociceptors also regulate inflammation and immunity. Previous studies have shown that DRG neurons participate in the stimulator of interferon genes (STING) mediated innate immune response and regulate nociception [5, 6].

STING is a strong inducer of type-I interferon (IFN-I) expression in immune cells following cancer, inflammation and tissue injury [7]. STING is an intracellular DNA sensor that plays a key role in the regulation of immunity and inflammation and promotes the elimination of pathogens and damaged host cells [8–10]. Generally, cyclic GMP–AMP synthase (cGAS ; GMP is guanosine monophosphate； AMP is adenosine monophosphate) detects and binds pathogenic DNA (exogenous DNA and endogenous DNA) in the cytoplasm. Upon binding to DNA, cGAS undergoes a conformational change to an active state and produces the second messenger cyclic GMP–AMP (cGAMP) [9, 11]. cGAMP acts as a second messenger to activate STING and drives STING translocation from the endoplasmic reticulum to perinuclear microsomes via the Golgi apparatus [12]. Subsequently, activated STING recruits and phosphorylates TANK-binding kinase 1 (TBK1), which further activates interferon regulatory factor 3 (IRF3) and nuclear factor κappa B (NF-κB), which enter the nucleus to induce the expression of IFN-I and proinflammatory factors such as IL-1β, IL-6 and TNF-α [7]. The cGAS–STING pathway has emerged as a potential mechanism underlying many inflammation-mediated pathophysiological processes, including tumorigenesis and neuroinflammation [13–15].

Moreover, previous studies have demonstrated that DRG neurons from pain models exhibit oxidative stress-related mitochondrial damage [16]. After mitochondrial stress, mitochondrial DNA (mtDNA) enters the cytoplasm, binds to intracellular DNA receptors such as STING and then triggers an autoimmune inflammatory response in DRG neurons, which may be an important initiating factor for chronic inflammatory responses [17]. Therefore, exploring the immune and inflammatory responses of DRG neurons to mitochondrial injury may shed new light on the mechanism of BCP.

Neuroinflammation plays an important role in the occurrence and development of BCP [18, 19]. In general, neuroinflammation is a physiological process that protects the body and promotes tissue regeneration and wound healing. However, long-term neuroinflammation may lead to the development of chronic pain [19, 20]. Accumulating evidence indicates that STING is involved in the production and maintenance of neuroinflammation and inflammatory pain [20–22]. A recent study in a spared nerve injury (SNI) model showed that the STING pathway participates in the regulation of neuropathic pain [23]. In addition, tumor-generated DNA mediates neuroinflammation and cancer-induced pain through STING-mediated activation of microglia [24]. However, it remains unclear whether the STING pathway induces neuroinflammation to regulate the development of BCP [20, 23, 24].

In this study, we assessed whether the STING pathway is a critical regulator of neuroinflammation and its possibility as a therapeutic target for BCP.

## Materials and methods

### Animals

Adult female Sprague–Dawley rats (6–8 weeks of age, Shanghai JieSiJie Laboratory Animals Co,. LTD, Shanghai, China) were used in this study. The animals were housed in a specific pathogen-free, temperature-controlled facility. The rats were housed at five per cage at a temperature of 24 ± 1 °C on a 12-h/12-h light–dark cycle and provided free access to food and water. All animal protocols were carried out following the guidelines of the International Association for the Study of Pain and were approved by the Animal Care and Use Committee of Shanghai Chest Hospital, Shanghai Jiao Tong University School of Medicine [permission no. KS (Y)21004].

### Cancer cell preparation

The Walker 256 rat mammary gland carcinoma cell line was purchased from American Type Culture Collection (ATCC, USA). As described previously, 0.5 mL (2 × 10^7^ cells/mL) of cancer cells was injected into the abdominal cavities of female Wistar rats weighing 70–80 g [25]. After 1 week, 2 mL of ascitic fluid was extracted and centrifuged for 5 min at 1000 rpm. Subsequently, the pellet was washed with 10 mL of phosphate-buffered saline (PBS, pH = 7.4) and centrifuged again for 5 min at 1000 rpm. After being resuspended in 1 mL of PBS, the cells were counted and diluted to a final concentration of 5 × 10^5^ cells/10 μL in PBS and kept on ice until injection. Animals in the sham group underwent the same procedures but were injected with an equal volume and density of heat-killed carcinoma cells instead of normal carcinoma cells.

### Rat BCP model

The rat BCP model was induced by injecting Walker 256 carcinoma cells into the bone marrow cavity of the rat tibia as previously described [26]. Briefly, female Sprague–Dawley rats weighing 150–200 g were anesthetized with pentobarbital (2%, 40 mg/kg). A 23-gauge needle was inserted into the lower third of the intramedullary canal of the right tibia to create a cavity for cell injection, and 10 μL of Walker 256 cells (5 × 10^5^ cells) (BCP group) was slowly injected into the bone cavity of the right tibia. The syringe was then removed, and the injection site was immediately closed with bone wax. Then, penicillin was applied to the wound. The rats in the sham group were subjected to the same procedures but were injected with an equal volume of heat-treated carcinoma cells instead of normal carcinoma cells. According to the experimental design, the rats were euthanized, and the tibias were collected for gross examination.

### Drug administration

The STING antagonist C-176 (HY-112906, purchased from MedChemExpress, New Jersey, USA) was dissolved in 200 μL of corn oil (Sigma–Aldrich, MO, USA) at final doses of 525 nM and 5250 nM for intraperitoneal (i.p.) injection, and a preparation containing 70 nM was injected intrathecally (i.t.) on the 10th day after tumor inoculation in the rat model according to instructions from MedChemExpress and the dose conversion formula between mice and rats based on body surface area [5]. Control rats received an equal volume of vehicle solution (*n* = 5 for each group). Then, 5250 nM of C-176 was repeatedly i.p. administered from days 10 to 16 after inoculation to test the long-term analgesic effect. Control rats were repeatedly i.p. administered an equal volume of vehicle solution (*n* = 5 for each group). Behavioral tests were performed 1 day before modeling and 1 day before drug injection to obtain baseline data and were also conducted 1 h, 2 h, 4 h, 24 h, 48 h, 72 h and 96 h after the first injection or 24 h after each repeated injection.

### Behavioral assays

#### Paw withdrawal mechanical threshold (PWMT) assay

Von Frey filaments were used to assess mechanical allodynia of the right hind paw as previously described [27]. Briefly, the animals were individually placed in an inverted ventilated cage with a metal mesh floor. Von Frey monofilaments (0.6, 1, 1.4, 2, 4, 6, 8, 10, and 15 g) were applied vertically to the plantar surface in the right hind paw of rats for 5 s with an interstimulus interval of 15 s. A vigorous paw withdrawal or paw flinching was considered as a positive response and each stimulus was applied for 5 times. The PWMT test followed the blind principle. The paw withdrawal mechanical frequency (PWMF) in response to each monofilament was calculated based on five applications. The PWMT was calculated as the force at which the PWMF was ≥ 60%; 15 g was recorded as the PWMT if the PWMF was < 60% for all filaments.

#### Limb use score (LUS) test

Movement-evoked pain was assessed by determining the limb use score as previously described [27]. The behavioral assays were performed in a blinded manner. The rats were allowed to move freely on a smooth, plastic table (50 cm × 50 cm), and limb use during free ambulation was scored on a scale of 4–0 (4, normal use; 3, slight limping; 2, clear limping; 1, no use of the limbs (partial); and 0, no use of the limbs (complete)).

### Bone histology

Rats were anesthetized with pentobarbital (2%, 40 mg/kg) and killed. The right tibia was removed from each rat, fixed in 4% PFA for 48 h and then decalcified in 10% EDTA for 3–4 weeks. The bones were rinsed, dehydrated, embedded in paraffin, and cut into 5-μm cross-sections using a rotary microtome (Reichert-Jung 820, Cambridge Instruments). Paraffin-embedded sections were stained with hematoxylin–eosin (H&E; Sigma, USA) to visualize the extent of tumor infiltration and bone destruction.

### Microcomputed tomography (Micro-CT)

Micro-CT analyses were performed on tibias from tumor-inoculated rats or sham-operated rats using a VivaCT 80 scanner with the 55-kVp source (Scanco, Southeastern, PA) as previously described [28]. 3D reconstruction images were acquired with Scanco Medical software. Quantification of micro-CT data was calculated for proximal tibias of rats. Statistic parameters included bone volume/total volume (BV/TV), connectivity density (Conn.D).

### Transmission electron microscopy (TEM)

Rats were anesthetized with pentobarbital (2%, 40 mg/kg) and killed, and the L3-L5 DRGs were isolated from rats and fixed with 4% paraformaldehyde (PFA) containing 1% glutaraldehyde in 0.1 M cacodylate buffer overnight at 4 °C as previously described [29]. Thereafter, the samples were washed with distilled water at room temperature. After dehydration with graded alcohol solutions, the samples were embedded in epoxy resin. Ultrathin sections (100 nm) were cut using a Leica Ultracut microtome, collected on copper grids, and stained with uranyl acetate and lead citrate with a Leica EM Stainer. Finally, the sections were examined with a JEM 1010 transmission electron microscope (JEOL Ltd., Japan) at an accelerating voltage of 100 kV to assess structural and morphological changes in mitochondria, such as mitochondrial swelling, and changes in mitochondrial cristae.

### Western blotting

Rats were anesthetized with pentobarbital (2%, 40 mg/kg) and killed, and the L3-L5 DRGs were then harvested, lysed, and centrifuged twice at 12,000 rpm for 20 min at 4 °C as previously described [22]. The protein concentration was measured with a bicinchoninic acid assay (Solarbio, China). Samples containing approximately 50 μg of protein were separated using 10% SDS–PAGE and then transferred onto polyvinylidene difluoride membranes (Solarbio, China). Next, the membranes were blocked with 5% bovine serum albumin in Tris-buffered saline [TBS; 50 mM Tris–HCl and 150 mM NaCl (pH 7.5)] for 2 h at room temperature and then incubated overnight at 4 °C with specific antibodies against the following: BAX (1:1000, Abcam, USA), STING (1:1000, Cell Signaling Technology, USA), P-TBK1/NAK (Ser172) (D52C2) (1:1000, Cell Signaling Technology, USA), TBK1/NAK (1:1000, Abcam, USA), P-IKK (Ser172) (D1B7) (1:1000, Cell Signaling Technology, USA), IKK (D61F9) (1:1000, Cell Signaling Technology, USA), P-p65 (Ser536) (93H1) (1:1000, Cell Signaling Technology, USA), p65 (D14E12) (1:1000, Cell Signaling Technology, USA), IL-1β (1:100, Santa Cruz Biotechnology, USA), IL-6 (1:1000, ABclonal, China), TNF-α (1:1000, ABclonal, China), and GAPDH (1:1000, ABclonal, China). Subsequently, the membranes were incubated with the appropriate horseradish peroxidase-conjugated secondary antibodies (1:2000, Cell Signaling Technology, USA) in TBST containing 5% BSA for 2 h at room temperature, and immunoreactive proteins were visualized using enhanced chemiluminescence (ChemiDoc XRS1, Bio–Rad, Hercules, CA). The intensities of the light-emitting bands were detected and quantified with Image Lab software (ImageJ, version 2.0.0-rc-69/1.52p, https://imagej.net/). The expression levels of BAX, STING, IL-1β, IL-6 and TNF-α were normalized to the expression level of GAPDH. The levels of P-TBK1, P-IKK, and P-NF-κB p65 were normalized to the levels of the corresponding total forms of these proteins.

### Enzyme‑linked immunosorbent assay analysis (ELISA)

The concentrations of IL-1β, IL-6 and TNF-α in the collected DRG samples were measured using rat IL-1β (ER9074, Wellbi Biological Technology, China) TNF-α (ER9113M, Wellbi Biological Technology, China) and IL-6 (ER9078M, Wellbi Biological Technology, China) ELISA kits [30]. In brief, six diluted standard protein (720, 360, 180, 90, 45, and 22.5 µg/L) in 50 µL buffer and 50 µL of blank control buffer were added into the 96-well plate in triplicates. Subsequently, a mixture of 10 µL of samples of the different groups and 40 µL of buffer were also added into the plate in triplicate. Then, the plate was incubated in a water bath for 30 min at 37 °C and washed 5 times. The color developing agents were added into each well and incubated at 37 °C in the dark for 10 min. Finally, the absorbance of each well was measured using a microplate reader at 450 nm after adding 50 µL of termination agent.

### RNA extraction and real-time quantitative PCR (RT–qPCR)

Rats were anesthetized with pentobarbital (2%, 40 mg/kg) and killed, and the DRGs were quickly removed and stored in liquid nitrogen as previously described [31]. Total RNA was isolated using RNA-Easy Isolation Reagent (Vazyme Biotech, China), and complementary DNA (cDNA) was generated using a cDNA Synthesis Kit (DBI Bioscience, Germany) according to the manufacturer’s instructions. RT–qPCR was carried out using SYBR Green (DBI Bioscience, Germany) and the StepOnePlus™ Real-Time PCR System (Applied Biosystems, Thermo Fisher Scientific, USA). Relative gene expression levels were calculated by the comparative cycle threshold (CT; 2^−ΔΔCt^) method. GAPDH was used as a housekeeping gene. The sequences of primers specific for STING and GAPDH were as follows:

STING: sense: 5′-CAGCCTGATGAGCCTTTGGATGAC-3′;

Antisense: 5′-GGACTGGACATGGCACAACTCTTC-3′;

GAPDH: sense: 5′-GGTGGACCTCATGGCCTACA-3′;

Antisense: 5′-CTCTCTTGCTCTCAGTATCCTTGCT-3′.

### Immunofluorescence (IF) staining

Rats were anesthetized with pentobarbital (2%, 40 mg/kg) and transcardially perfused with 10 mL of 4 °C PBS and 10 mL of 4% PFA as previously described [32]. The DRGs were carefully removed, placed in 4% PFA overnight, and stored at 4 °C. Then, the samples were transferred to 30% sucrose and stored for varying lengths of time (minimum of 1 day). The samples were later frozen separately in OCT compound. The samples were cut at a 10-μm thickness using a cryostat (CM1850-1-1, Leica, Germany) and mounted on slides. All samples were thoroughly rinsed in PBS to remove any residual OCT compound. The samples were then blocked in 0.3% Triton-X 100 and 5% normal donkey serum in PBS for 1 h. The samples were incubated overnight with primary antibodies in 0.3% Triton-X 100 and 5% NDS in PBS. The following primary antibodies were used: rabbit anti-STING antibody (19851-1-AP, 1:200, Proteintech, USA) and mouse anti-TuJ1 antibody (#60052, 1:200, STEMCELL Technologies, Canada). Then, the samples were incubated with a mixture of goat anti-mouse (Alexa Fluor 488-conjugated (green), Cell Signaling Technology, USA) and goat anti-rabbit (Alexa Fluor 594-conjugated (red), Cell Signaling Technology, USA) antibodies. The next day, the samples were rinsed in PBS and incubated for 1 h with secondary antibodies in PBS. The samples were then rinsed in PBS and coverslipped with Antifade Mounting Medium with DAPI (Beyotime, China). The slides were observed by laser scanning confocal microscopy, and image acquisition and analysis were performed using a TCS SP8 confocal microscope (Leica Microsystems, Germany). Quantitative analysis of the average fluorescence intensity and the number of positively stained neurons was performed in a blinded manner using ImageJ software (ImageJ, version 2.0.0-rc-69/1.52p, https://imagej.net/). The mean fluorescence intensity (mean gray value) was calculated as the integrated density (IntDen)/area. The average fluorescence intensity is expressed in arbitrary units (a.u.). To determine the cellular distribution of STING, double IF staining of TuJ1 and STING in the DRG of normal rats and tumor-bearing rats was conducted.

### Statistics

The data are expressed as the mean ± SD. The ImageJ and GraphPad 8.0 software programs were used to organize the data, generate the figures, and perform the statistical tests. All behavioral tests and other experiments were performed in a blinded manner. Two-way repeated-measures ANOVA followed by Tukey’s post hoc test was used to analyze the behavioral data. For the other data, comparisons between two groups were made using two-tailed unpaired Student’s *t* tests, and comparisons among more than three groups were made using one-way ANOVA followed by Tukey’s post hoc test. Differences for which the *p* value was smaller than 0.05 were considered statistically significant.

## Results

### Tumor-bearing rats exhibited decreased pain thresholds and significant bone destruction

**Fig. 1:**
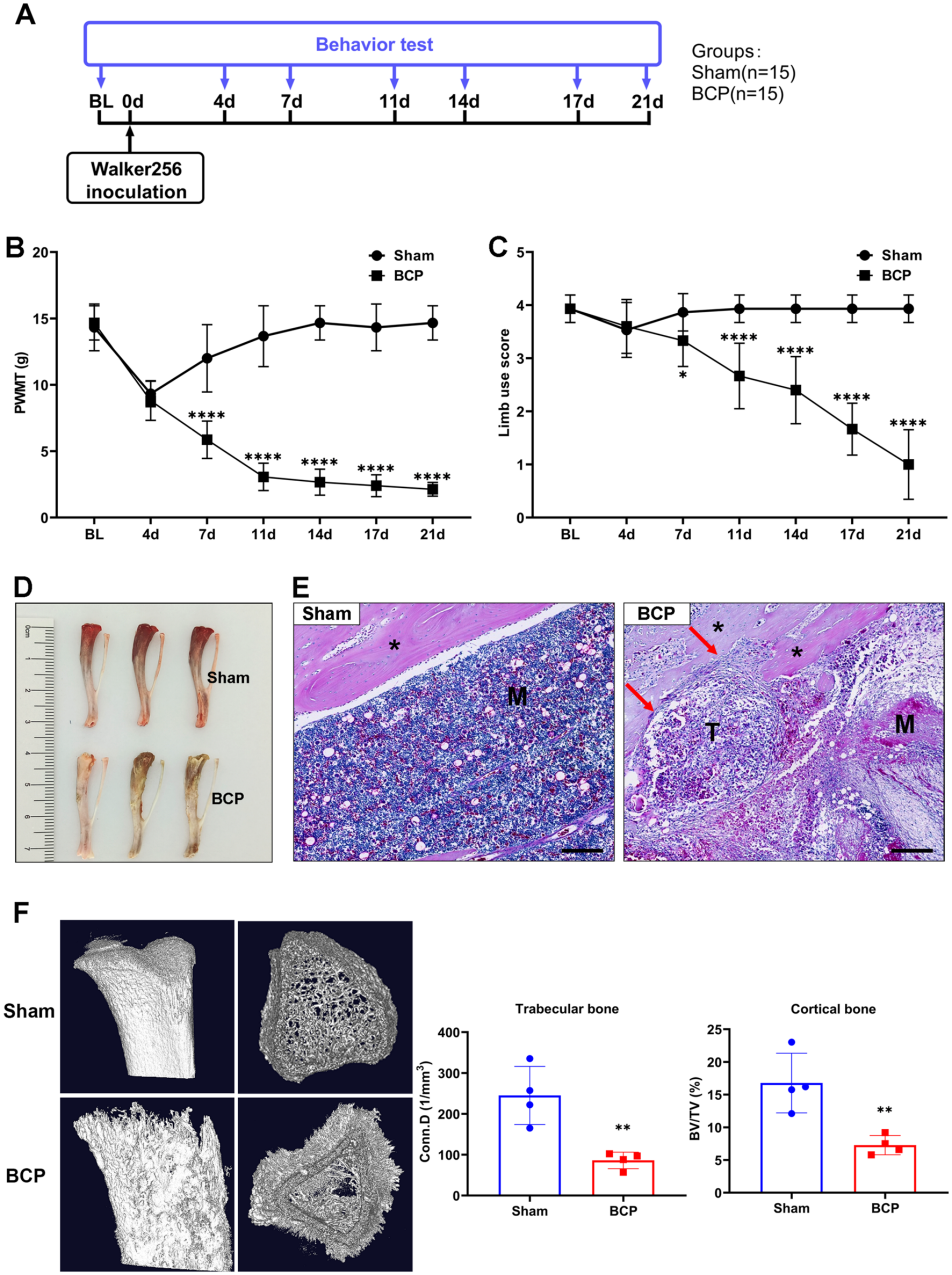
Tumor-bearing rats exhibited decreased pain thresholds and significantly increased bone destruction. **A** Experimental scheme of the protocols used for BCP model establishment and behavioral analysis. **B** The paw withdrawal mechanical threshold (PWMT) of the sham and BCP groups. ****p < 0.0001. *n* = 15 per group. **C** The limb use score of the sham and BCP groups in the pain behavior tests. ****p < 0.0001. *n* = 15 per group. **D** Anatomic structure of the tibias of rats in the sham and BCP groups. **E** Representative hematoxylin and eosin (H&E) staining images of bones from the sham and BCP groups. Scale bar = 200 μm. *The cortex of bone. T tumor, *M* the matrix of bone. The red arrows indicate areas of significant bone destruction. **F** Representative micro-CT images of bones from the sham and BCP groups. Morphometric parameters from micro-CT including BV/TV and Conn.D. *Conn.D* connectivity density, *BV/TV* bone volume/total volume. **p < 0.01. *n* = 4 per group (color figure online)

Behavioral assays were performed on days 0, 4, 7, 11, 14, 17, and 21 after intratibial inoculation of Walker 256 mammary gland carcinoma cells (Fig. 1A). As shown in Fig. 1B and C, during the first 4 days after intratibial injection, PWMT and LUS were significantly reduced in both groups, possibly because the needle cavities have not yet fully filled. During the next 4 to 7 days, the two groups showed different pain behaviors. Sham-operated rats gradually recovered, whereas BCP rats became more and more painful as tumors grew, persisting for at least 21 days (Fig. 1B, C). Then, the rats were euthanized, and their tibias were isolated. The tibias from the rats with bone cancer were more swollen and uneven compared to that in the tibias from the sham rats (Fig. 1D). H&E staining of tibia sections showed tumor cells replaced normal bone marrow cells and filled the bone marrow cavity to invade the bone cortex, leading to obvious destruction of the bone cortex (Fig. 1E). Micro-CT images showed that bone cortex destruction and bone trabecular reduction appeared on the tibias of rats in the BCP group. There was a significant reduction in trabecular bone connectivity density (Conn.D) as well as in cortical bone volume/total volume (BV/TV) based on quantitative evaluation of the data (Fig. 1F). Collectively, these results indicated that the BCP model was successfully established.

### Mitochondrial stress and STING expression levels in the DRG were increased in BCP rats

**Fig. 2:**
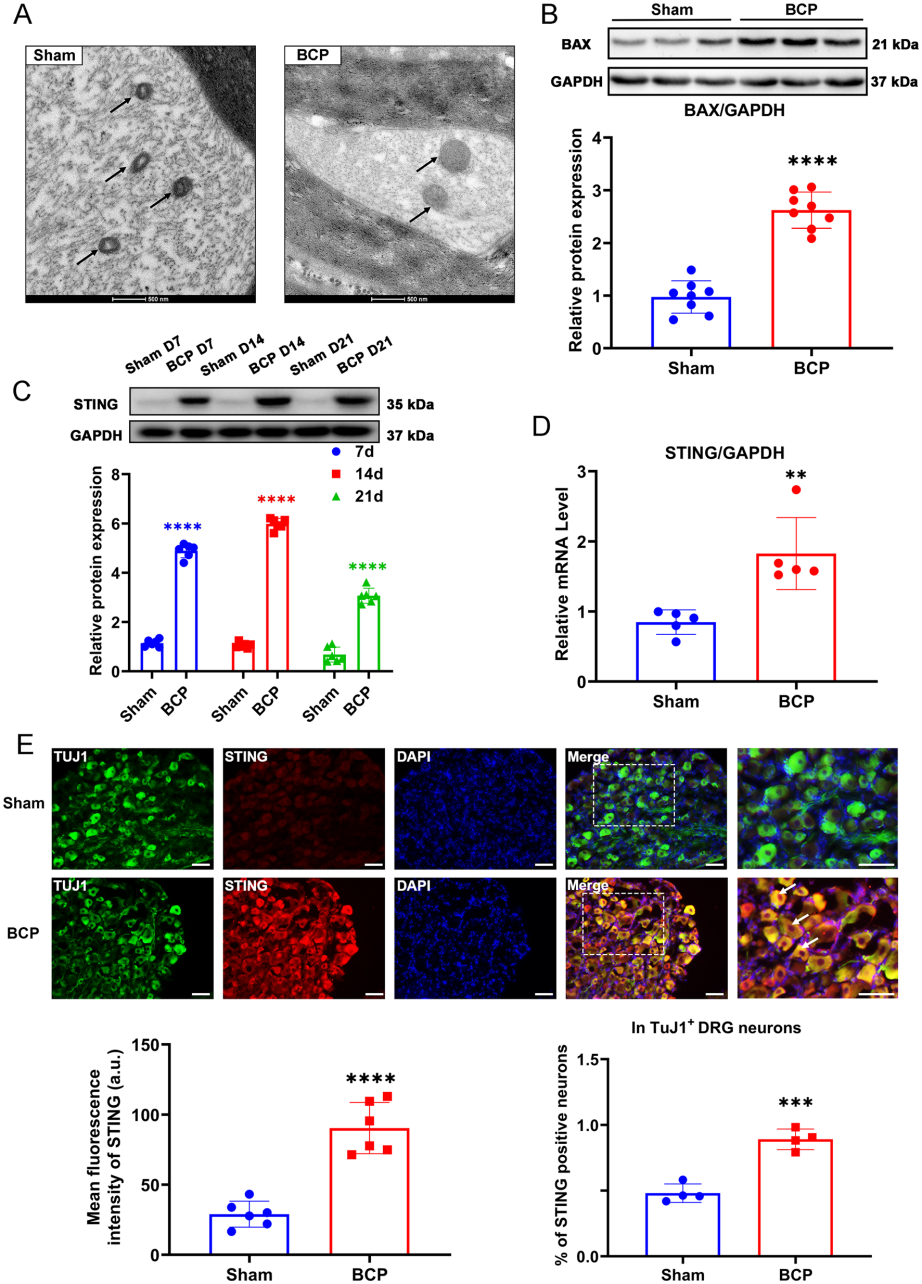
Mitochondrial stress and STING expression were observed in the BCP model at the indicated time points. **A** Mitochondrial morphology of DRGs from the sham and BCP groups viewed under an electron microscope. Scale bar = 500 nm. The black arrows indicate mitochondria. **B** Western blotting analysis of BAX expression in L3–L5 DRGs. Representative protein bands are shown on the top of the corresponding histogram. *n* = 8 per group. **C** Protein levels of STING at the indicated time points between Sham and BCP. *n* = 6 per group. **D** RT–qPCR analysis of STING expression in L3–L5 DRGs on day 14. *n* = 5 per group. **E** Representative IF images of STING (red), TuJ1 (green) and DAPI (blue) in the DRGs of sham and BCP rats. The white arrows indicate typical co-stained cells. Scale bar = 50 μm. Mean fluorescence intensity of STING (left) and the percentage of STING-positive neurons in TuJ1^+^ DRG neurons (right) after the injection of C-176. *n* = 4 per group. Data are expressed as mean ± SEM. *p < 0.05, **p < 0.01, ***p < 0.001. ****p < 0.0001 (color figure online)

As shown in Fig. 2A, mitochondria showed severe structural defects, including mitochondrial swelling and disappearance of the structural ridge, under electron microscopy. It is thought that BAX is a proapoptotic factor that promotes mitochondrial stress and apoptosis. In BCP rats, BAX expression was higher than in sham-operated rats, suggesting that mitochondrial stress may play a role (Fig. 2B). The protein level of STING in the DRG was significantly increased from day 7 to day 21 after bone cancer induction in the tumor-bearing rats compared with Sham group rats at the corresponding time point and peaked at day 14 (Fig. 2C). Consistently, STING mRNA transcripts detected by RT-PCR (Fig. 2D), and protein levels detected by IF (Fig. 2E) were elevated In the BCP group. Further, double IF staining showed that STING and TuJ1 colocalized in DRG neurons with a higher percentage of STING-positive cells in TuJ1^+^ DRG neurons in BCP group than in Sham group, indicating a significant increase in the number of DRG neurons that expressed STING protein in the BCP group (Fig. 2E). Taken together, our findings showed that the BCP rats exhibited mitochondrial stress and increased expression levels of STING in the DRG compared to those of the sham rats, suggesting that mitochondrial stress may be a factor involved in the activation of STING.

### The inflammatory pathway downstream of STING was activated in the BCP model

**Fig. 3:**
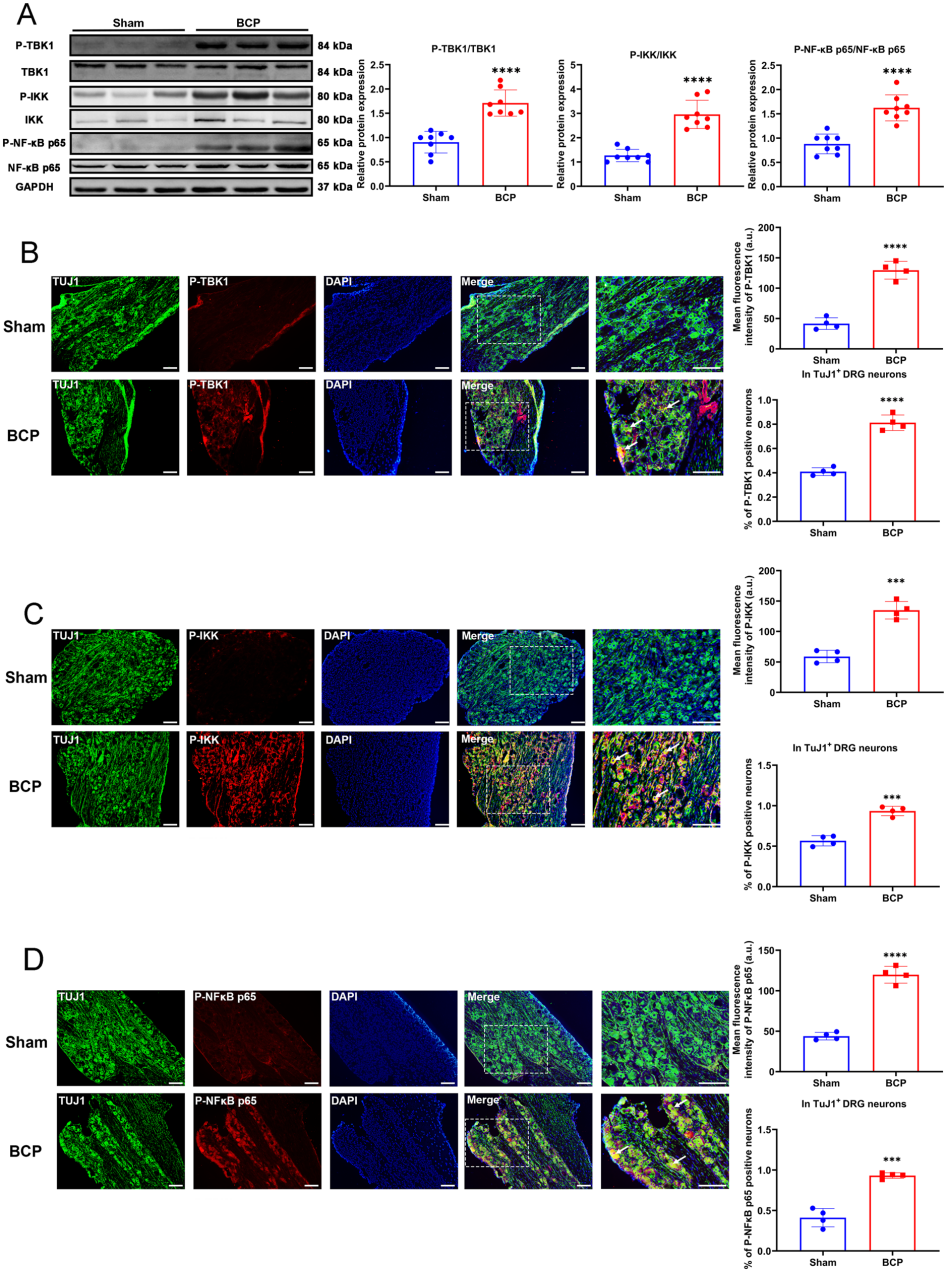
Expression of downstream signaling pathway of STING. **A** The expression of P-TBK1/TBK1, P-IKK/IKK and P-NF-κB p65/NF-κB p65 in the L3-L5 DRGs of rats in the sham and BCP groups on day 14 were determined by western blotting. Representative protein bands were on the right. GAPDH was detected as an internal control. *n* = 8 per group. The expression and cellular localization of P-TBK1 (**B**), P-IKK (**C**) and P-NF-κB p65 (**D**) in the L3-L5 DRGs of rats in the sham and BCP groups on day 14 were determined by immunofluorescence. Representative IF images of P-TBK1 (red), P-IKK (red), P-NF-κB p65 (red), TuJ1 (green) and DAPI (blue) are shown on the right. The white arrows indicate typical co-stained cells. Scale bar = 50 μm. Mean fluorescence intensity (upper) and percentage of positive neurons in TuJ1^+^ DRG neurons (down) are shown on the left. Data are expressed as mean ± SEM. ****p < 0.0001. *n* = 4 per group (color figure online)

**Fig. 4:**
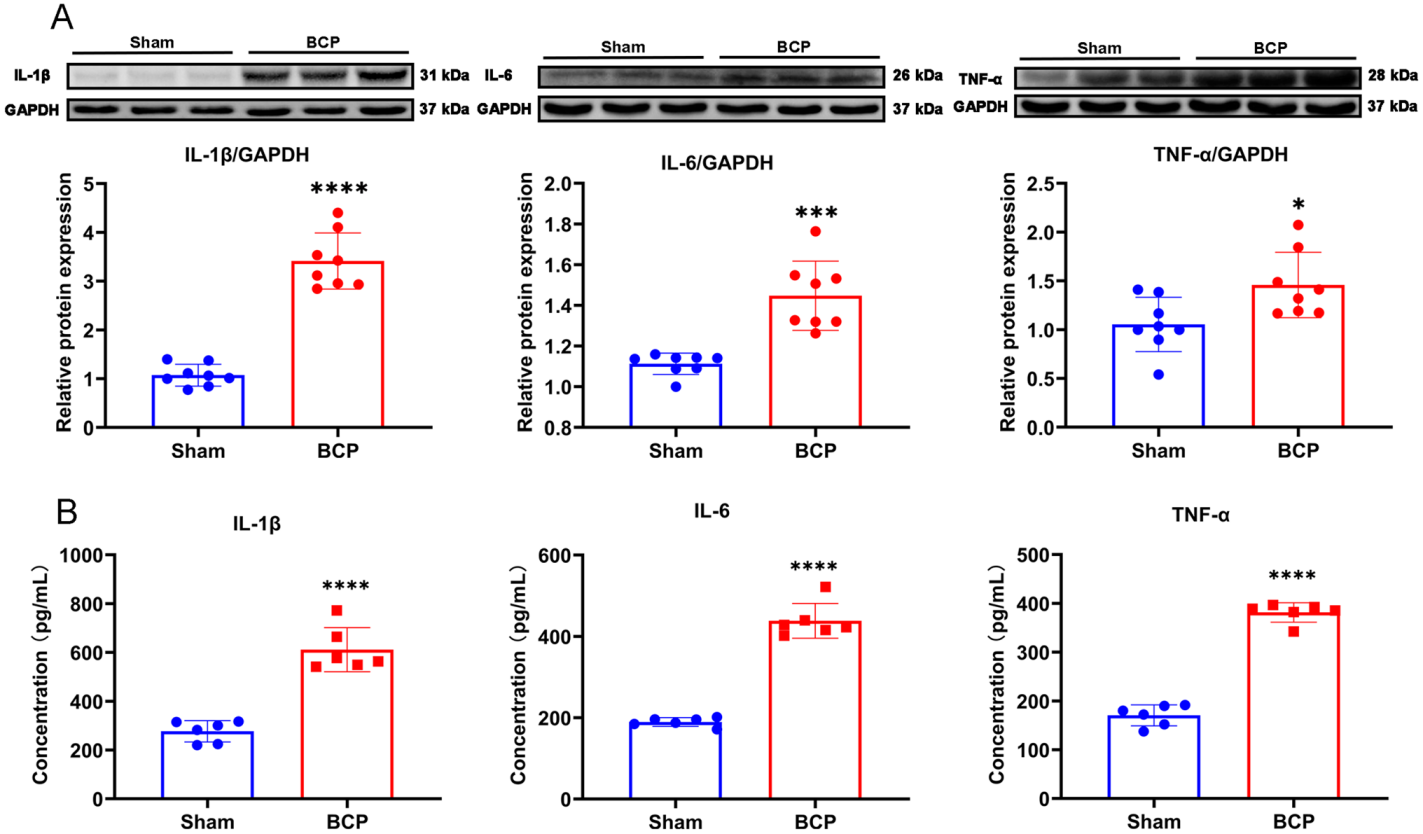
The downstream proinflammatory factors were significantly increased in DRG of BCP rats. **A** Western blotting analysis of IL-1β, IL-6 and TNF-α in L3-L5 DRGs of BCP rats on day 14. Representative protein bands are shown on the top of the corresponding histogram. *n* = 8 per group. **B** The concentration of IL-1β, IL-6 and TNF-α in DRG were measured using ELISA kits. Data are expressed as mean ± SEM. *p < 0.05, ***p < 0.001, ****p 0.0001. *n* = 6 per group (color figure online)

Western blotting showed that the levels of P-TBK1, P-IKK, and P-NF-κB p65 in the DRG neurons were significantly increased in BCP rats, suggesting activation of the STING/TBK1/NF-κB pathway (Fig. 3A). In addition, we also used immunofluorescence staining to detect P-TBK1, P-IKK, and P-NF-κB p65 protein expression and cellular localization (Fig. 3B–D). The results showed that P-TBK1, P-IKK, and P-NF-κB p65 protein was mainly expressed and activated in DRG neurons, and significantly increased in expression in DRG of BCP rats (Fig. 3B–D). Concurrently, the results of western blotting (Fig. 4A) and ELISA (Fig. 4B) showed that the protein levels of the downstream inflammatory cytokines IL-1β, IL-6 and TNF-α in the DRG were also increased in the BCP rats, revealing the activation of neuroinflammation. These results showed that activation of the STING signaling pathway in DRG neurons might be associated with peripheral neuroinflammation.

### C-176 ameliorated BCP by inhibiting STING expression and neuroinflammation

**Fig. 5:**
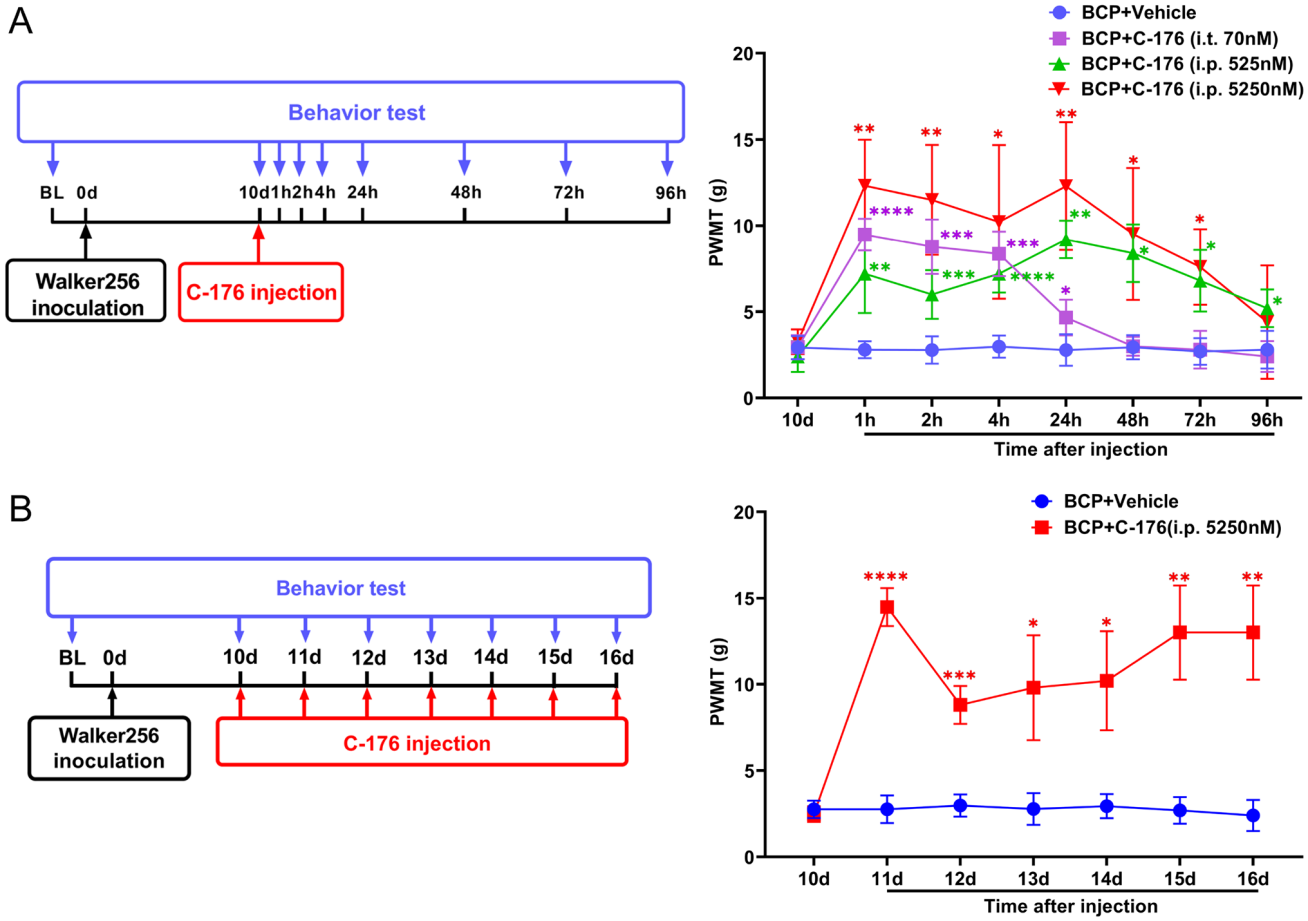
Alleviation of bone cancer-induced pain after injection of C-176. **A** Experimental scheme of the protocol used for single intrathecal or intraperitoneal injection of C-176 and assessments of changes in the PWMT at the indicated time points after injection. *n* = 5 per group. **B** Experimental scheme of the protocol used for intraperitoneal injection of C-176 every day for 6 days and assessments of changes in the PWMT at the indicated time points after injection. Data are expressed as mean ± SEM. *p < 0.05. **p < 0.01. ***p < 0.001. ****p < 0.0001. *n* = 5 per group. *i.t.* intrathecal injection, *i.p.* intraperitoneal injection (color figure online)

**Fig. 6:**
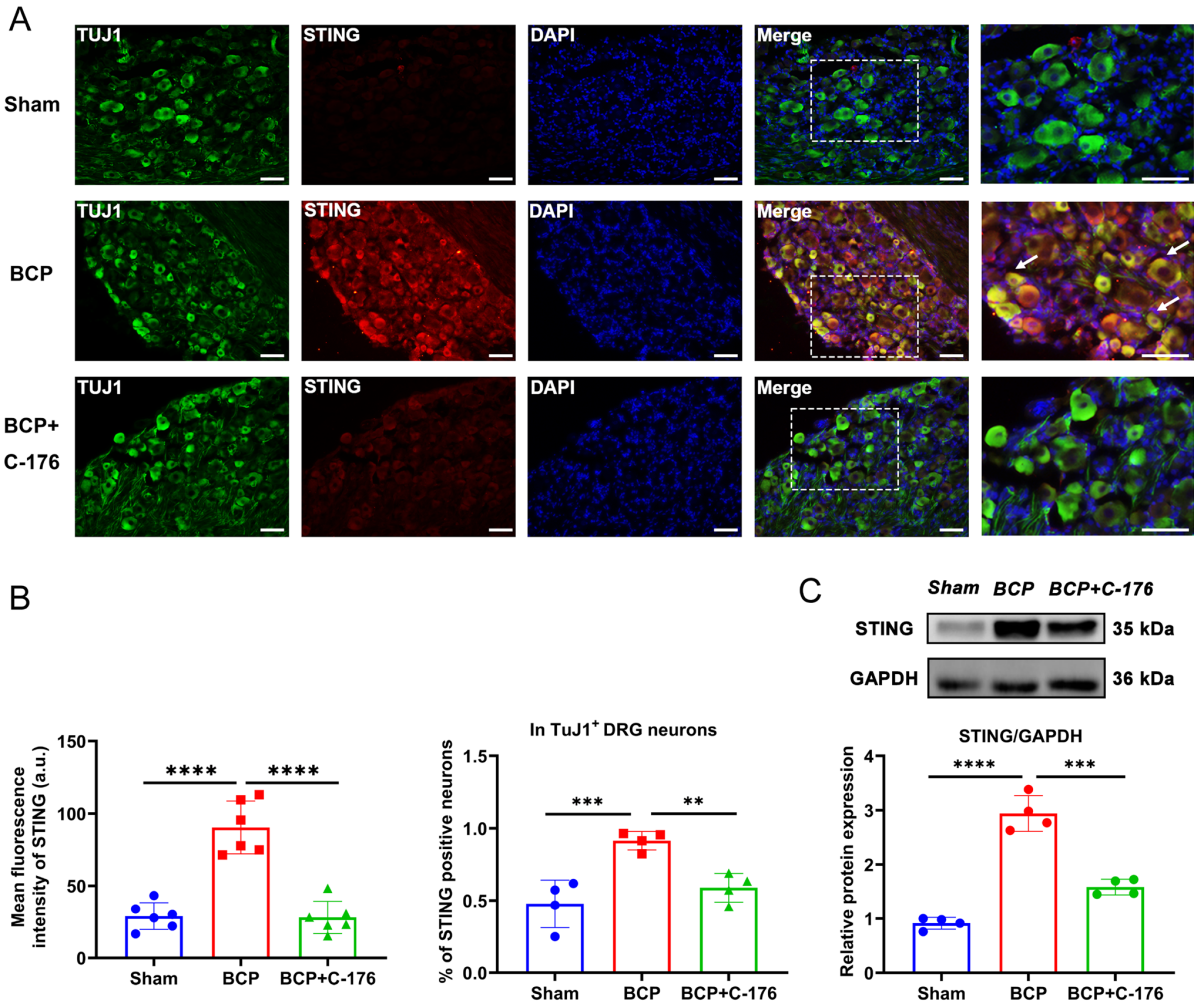
Expression level of STING in the DRG after intraperitoneal injection of C-176. **A** Representative IF images of STING (red), TuJ1 (green) and DAPI (blue) in the DRG after the injection of C-176. The white arrows indicate typical co-stained cells. Scale bar = 50 µm. **B** Mean fluorescence intensity of STING (left) and the percentage of STING-positive neurons in TuJ1^+^ DRG neurons (right) after the injection of C-176. *n* = 6 per group. **C** Western blotting analysis of STING expression in L3-L5 DRGs. Representative protein bands are shown on the top of the corresponding histogram. Data are expressed as mean ± SEM. **p < 0.01, ***p < 0.001. ****p < 0.0001. *n* = 4 per group (color figure online)

**Fig. 7:**
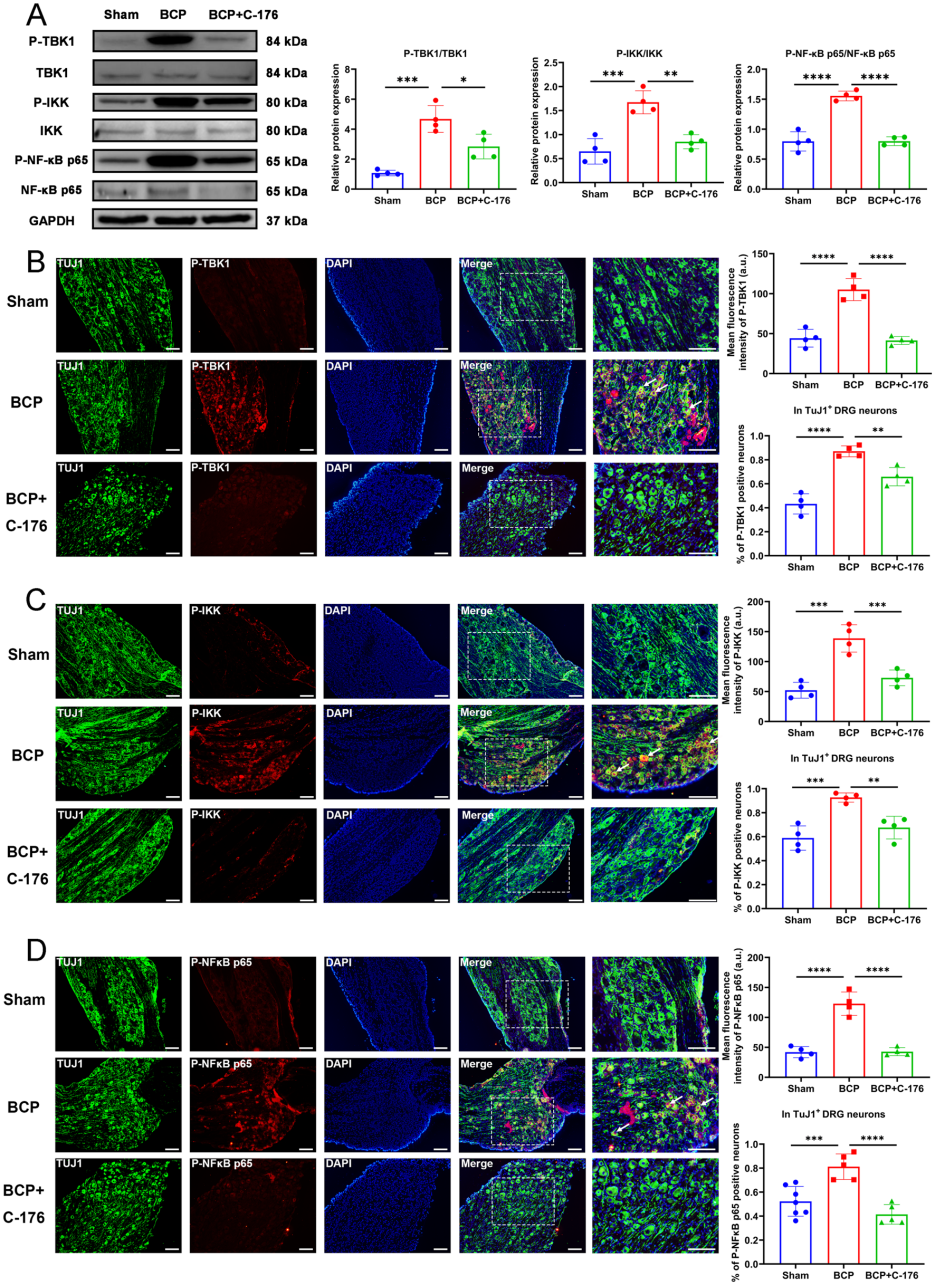
Expression of downstream signaling pathway of STING after the intraperitoneal injection of C-176. **A** The expression of P-TBK1/TBK1, P-IKK/ IKK and P-NF-κB p65/NF-κB p65 in the L3–L5 DRGs of rats after the injection of C-176 were determined by western blotting. Representative protein bands were on the right. GAPDH was detected as an internal control. The expression and cellular localization of P-TBK1 (**B**), P-IKK (**C**) and P-NF-κB p65 (**D**) in the L3-L5 DRGs of rats in the DRGs were determined by immunofluorescence. Representative IF images of P-TBK1 (red), P-IKK (red), P-NF-κB p65 (red), TuJ1 (green) and DAPI (blue) are shown on the right. The white arrows indicate typical co-stained cells. Scale bar = 50 μm. Mean fluorescence intensity (upper) and percentage of positive neurons in TuJ1 + DRG neurons (down) are shown on the left. Data are expressed as mean ± SEM. *p < 0.05, **p < 0.01, ***p < 0.001, ****p < 0.0001. *n* = 4 per group (color figure online)

**Fig. 8:**
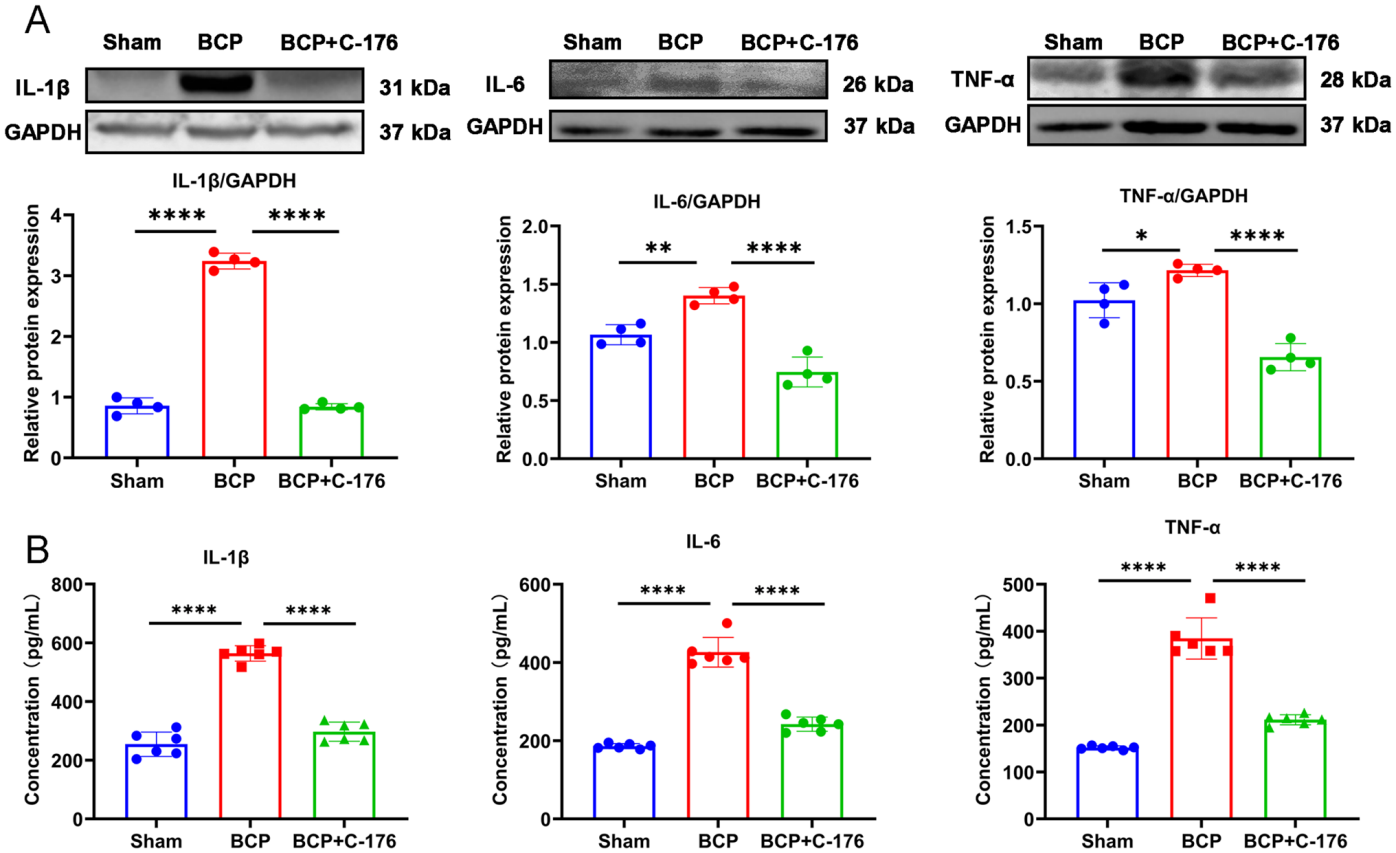
Expression of proinflammatory factors of DRG after the intraperitoneal injection of C-176. **A** Western blotting analysis of IL-1β, IL-6 and TNF-α in L3–L5 DRGs of BCP rats after the intraperitoneal injection of C-176. Representative protein bands are shown on the top of the corresponding histogram. *n* = 4 per group. **B** The concentration of IL-1β, IL-6 and TNF-α in DRG were measured using ELISA kits. Data are expressed as mean ± SEM. *n* = 6 per group. *p < 0.05, **p < 0.01, ****p 0.0001 (color figure online)

A single intraperitoneal injection of C-176 rapidly relieved BCP in a dose-dependent manner within 1 h, and this effect lasted for at least 72 h. Consistently, intrathecal injection of 70 nmol C-176 showed analgesic effect, lasting for only 24 h (Fig. 5A). Further, repeated intraperitoneal administration of 5250 nmol of C-176 had a sustained analgesic effect without obvious drug tolerance (Fig. 5B). Western blotting and IF staining results showed that administration of C-176 significantly reduced the expression levels of STING in neurons of DRG in BCP rats (Fig. 6). The bands of western blotting indicated the TBK1, IKK and NF-κB p65 protein levels were decreased after the administration of C-176 (Fig. 7A). In DRG, immunofluorescence staining revealed that p-TBK1, P-IKK and p-NF-κB p65 were significantly upregulated in BCP rats and co-stain with TUJ1^+^ neurons, which were reversed by C-176 administration (Fig. 7B–D). Then, we detected the protein levels of the downstream proinflammatory factors (IL-1β, IL-6 and TNF-α), the western blot and ELISA results showed that C-176 treatment could reversed the increase of proinflammatory factors (Fig. 8). These results suggested that C-176 may relieve BCP by inhibiting the STING/TBK1/NF-κB pathway and decreasing the production of downstream inflammatory factors.

## Discussion

**Fig. 9:**
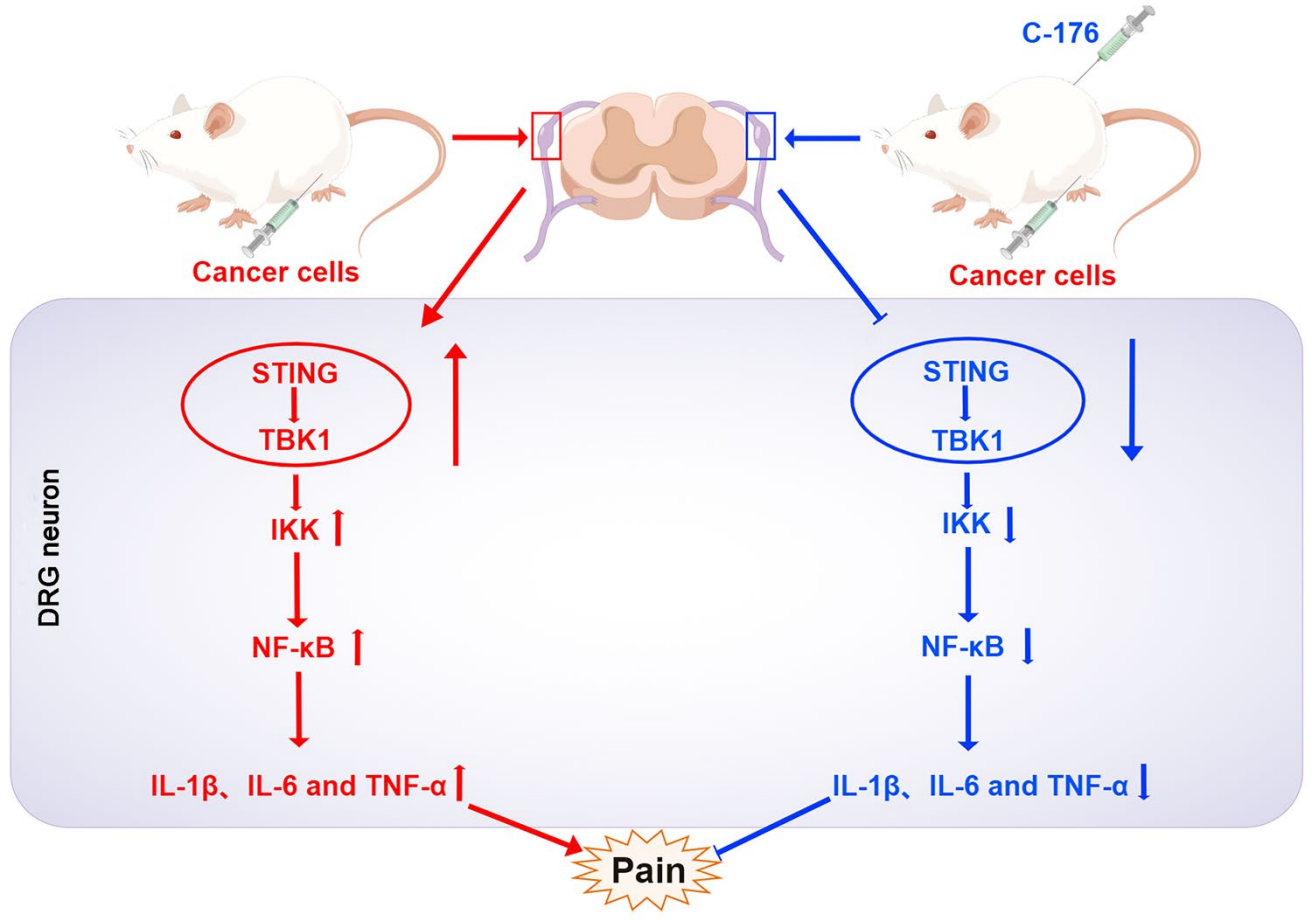
Schematic summarizing the mechanism by which STING promotes BCP. The expression level of STING in the DRG was significantly increased in BCP rats, sequentially activating the TBK1/IKK/NF-κB/proinflammatory factor axis to promote neuroinflammation and eventually induce enhanced BCP. After intraperitoneal injection of C-176, the level of STING in the BCP rats was decreased, and the TBK1/IKK/NF-κB/proinflammatory factor signaling pathway was then inhibited. Therefore, STING inhibition in the DRG relieved peripheral sensitization, which ultimately alleviated BCP. This figure was drawn with Figdraw (www.figdraw.com) (color figure online)

Here, we demonstrate that STING activation plays a vital role in neuroinflammation and BCP development. Specifically, we observed that STING was highly expressed in DRG neurons from BCP rats, consistent with the aggravation of pain behaviors. Intrathecal or intraperitoneal injection of the selective STING inhibitor C-176 alleviated hyperalgesia in BCP rats. Furthermore, DRG neurons from the BCP rats exhibited mitochondrial stress with an increased level of BAX, which indicated that the leakage of mtDNA from damaged mitochondria may be a STING activation trigger. After STING activation, IL-1β, IL-6, and TNF-α expression levels in the DRG were increased in the BCP model via the STING/TBK1/IKK/NF-κB pathway. To our knowledge, our research reveals for the first time that STING activation in DRG neuron drives bone cancer pain via the TBK1/IKK/NF-κB/proinflammatory factor signaling pathways, which could reverse by C-176 (Fig. 9).

It was previously believed that neuroinflammation was exclusively caused by immunological cells, such as microglia and astrocytes [33, 34]. However, recent studies have found that neurons can also produce inflammatory factors, which play an important role in neuroinflammation [5]. Our study is unique in that it is the first study to demonstrate time-dependent increased expression of STING in primary nociceptive neurons in the DRG of BCP rats. Various methods were employed to administer STING inhibitor C-176 to further determine STING’s role in BCP. On the 10th day after tumor inoculation, a single intraperitoneal or intrathecal injection of C-176 can relieve pain significantly. In the 10-to-16-day period after repeated intraperitoneal administration of C-176, a sustained analgesic effect was seen without any signs of drug tolerance. Collectively, STING in DRG nociceptive neurons may be responsible for peripheral sensitization related to cancer-induced bone pain, which can be relieved by administration of C-176. This study found some inconsistent findings regarding the role of STING in chronic pain in comparison to previous studies [5, 35]. In a study conducted by Wang et al., DMXAA relieved BCP in mice by increasing IFN-I production, focusing on a relatively early stage of bone cancer (3rd and 7th days after tumor inoculation). The focus of our study was primarily on the mid-late stage of bone cancer in BCP rats (10 days after BCP induction) [36]. It is possible that STING might play different roles at different stages of bone cancer, and this apparent discrepancy may be associated with different animal models, tumor stages, and treatments. In a mouse SNI model, C-176 relieved pain for at least four hours, regardless of whether it was administered early or late [23]. Notably, in our study, intraperitoneal injections produced a greater analgesic effect and lasted longer than intrathecal injections. Since intraperitoneally injected C-176 was applied systemically, it cannot be ruled out that other sites might participate in the regulation of pain.

The STING signaling pathway primarily activates IRF3 signaling and/or NF-κB signaling [37]. Our study found that STING and downstream signaling pathway proteins such as P-TBK1, P-IKK, and P-NF-κB p65 were upregulated in DRG of BCP rats, along with an increase in proinflammatory cytokines (IL-1β, IL-6, and TNF-α), which may promote BCP in the middle and late stages of bone metastasis. Further, C-176 significantly reduced the upregulation of P-TBK1, P-IKK, and P-NF-κB p65 in the DRG of BCP rats. A study by Tian et al. also found a close relationship between hyperalgesia and inflammatory response caused by STING activation [38].

Notably, we observed a significant change in the morphology of mitochondria in the DRG of BCP rats, and the protein level of BAX was obviously increased. Mitochondria are the main sites of intracellular oxidative phosphorylation and ATP production and regulate various biological processes in cells [39, 40]. When harmful stimuli from the external environment cause damage, mitochondria undergo aberrant structural and functional changes, resulting in mitochondrial stress [41, 42]. A large body of literature indicates that mtDNA released after mitochondrial stress is involved in the activation of STING and the production of proinflammatory factors [43–45]. Additionally, mitochondrial stress is closely related to the progression of cancer [46, 47]. Specifically, the BAX-mediated formation of pores in the mitochondrial outer membrane increases the permeability of the inner membrane, resulting in mtDNA efflux [48, 49]. Overall, our findings indicate that mtDNA may be released from mitochondria into the cytoplasm through BAX pores to activate STING and mediate neuroinflammation. However, this hypothesis needs to be further confirmed.

As the expression of STING was significantly increased in the BCP rats, we assessed the therapeutic effect of the STING inhibitor C-176 [35, 50]. The main effect of C-176 is to bind covalently to Cys-91 of STING, abrogating activation-induced palmitoylation which is crucial for its assembly into polymer complexes in Golgi apparatus, and in turn, it is used to recruit downstream signaling factors. Recent studies have shown that C-176 pretreatment can also inhibit STING expression dose-dependently [23, 51]. Our results were consistent with their results that C-176 can inhibit the expression of STING, but the mechanism is still unclear. The mechanism of BCP is very complex and involves a variety of immune and inflammatory signaling pathways. There are still some limitations of our study. First, we observed severe mitochondrial defects in the DRG of BCP mice, but we did not carry out a quantitative analysis of cytosolic mtDNA due to the limitations of the experimental conditions. Alternatively, the expression level of BAX was assessed. According to previous studies, an increase in the level of BAX expression is correlated with the formation of mitochondrial outer membrane pores, which may indirectly reflect mtDNA leakage in the cytoplasm [52]. Second, we found that the proinflammatory pathway downstream of STING was significantly activated, but activation of the middle steps in this pathway was not verified with inhibitors. Nevertheless, we demonstrated by administration of the STING inhibitor C-176 that the activation of STING is closely related to the production of proinflammatory factors. Third, the mechanism upstream of mitochondrial damage caused by BCP needs to be further explored. We speculate that tumor cells in the bone tumor microenvironment may release some nitrogen oxides, which are absorbed by afferent nerves and then transmitted to DRG neurons to trigger the oxidative stress response, causing mitochondrial damage. We will explore this hypothesis in depth.

## Conclusion

Our results indicate that the activation of STING in DRG neurons and proinflammatory factors downstream of STING led to neuroinflammation and peripheral pain sensitization in a rat model of BCP. Activation of the STING pathway may be related to mitochondrial stress and mtDNA leakage in DRG neurons. Therapeutic strategies targeting this pathway may be methods to prevent or treat BCP.

## Data Availability

The data used and/or analyzed during the current study are available from the corresponding author on reasonable request.

## Abbreviations

STING: Stimulator of interferon genes
BCP: Bone cancer pain
DRG: Dorsal root ganglia
BAX: BCL2-associated X
TBK1: TANK-binding kinase 1
NF-κB: Nuclear factor-kappa B
mtDNA: Mitochondrial DNA
IFN-1: Type-I interferon
cGAS: Cyclic GMP–AMP synthase
dsDNA: Double-stranded DNA
cGAMP: Cyclic GMP–AMP
IRF3: Interferon regulatory factor 3
SNI: Spared nerve injury
PWMT: Paw withdrawal mechanical threshold
i.p.: Intraperitoneal injection
i.t.: Intrathecal injection
PWMF: Paw withdrawal mechanical frequency
LUS: Limb use score
Micro-CT: Microcomputed tomography
TEM: Transmission electron microscopy
ELISA: Enzyme-linked immunosorbent assay analysis
PFA: Paraformaldehyde
TBS: Tris-buffered saline
RT-qPCR: RNA extraction and real-time quantitative PCR
cDNA: Complementary DNA
IntDen: Integrated density
a.u.: Arbitrary units
PRRs: Pattern recognition receptors
TLRs: Toll-like receptors
PAMPs: Pathogen-associated molecular patterns
DAMPs: Damage-associated molecular patterns
